# Structure/Function Studies Involving the V3 Region of the HIV-1 Envelope Delineate Multiple Factors That Affect Neutralization Sensitivity

**DOI:** 10.1128/JVI.01645-15

**Published:** 2015-12-30

**Authors:** Susan Zolla-Pazner, Sandra Sharpe Cohen, David Boyd, Xiang-Peng Kong, Michael Seaman, Michel Nussenzweig, Florian Klein, Julie Overbaugh, Max Totrov

**Affiliations:** a Veterans Affairs New York Harbor Healthcare System, New York, New York, USA; b Departments of Pathology and Biochemistry, New York University School of Medicine, New York, New York, USA; c Fred Hutchinson Cancer Research Center, Seattle, Washington, USA; d Beth Israel Deaconess Medical Center, Boston, Massachusetts, USA; e The Rockefeller University, New York, New York, USA; f Molsoft, L.L.C., San Diego, California, USA

## Abstract

Antibodies (Abs) specific for the V3 loop of the HIV-1 gp120 envelope neutralize most tier 1 and many tier 2 viruses and are present in essentially all HIV-infected individuals as well as immunized humans and animals. Vaccine-induced V3 Abs are associated with reduced HIV infection rates in humans and affect the nature of transmitted viruses in infected vaccinees, despite the fact that V3 is often occluded in the envelope trimer. Here, we link structural and experimental data showing how conformational alterations of the envelope trimer render viruses exceptionally sensitive to V3 Abs. The experiments interrogated the neutralization sensitivity of pseudoviruses with single amino acid mutations in various regions of gp120 that were predicted to alter packing of the V3 loop in the Env trimer. The results indicate that the V3 loop is metastable in the envelope trimer on the virion surface, flickering between states in which V3 is either occluded or available for binding to chemokine receptors (leading to infection) and to V3 Abs (leading to virus neutralization). The spring-loaded V3 in the envelope trimer is easily released by disruption of the stability of the V3 pocket in the unliganded trimer or disruption of favorable V3/pocket interactions. Formation of the V3 pocket requires appropriate positioning of the V1V2 domain, which is, in turn, dependent on the conformation of the bridging sheet and on the stability of the V1V2 B-C strand-connecting loop.

**IMPORTANCE** The levels of antibodies to the third variable region (V3) of the HIV envelope protein correlate with reduced HIV infection rates. Previous studies showed that V3 is often occluded, as it sits in a pocket of the envelope trimer on the surface of virions; however, the trimer is flexible, allowing occluded portions of the envelope (like V3) to flicker into an exposed position that binds antibodies. Here we provide a systematic interrogation of mechanisms by which single amino acid changes in various regions of gp120 (i) render viruses sensitive to neutralization by V3 antibodies, (ii) result in altered packing of the V3 loop, and (iii) activate an open conformation that exposes V3 to the effects of V3 Abs. Taken together, these and previous studies explain how V3 antibodies can protect against HIV-1 infection and why they should be one of the targets of vaccine-induced antibodies.

## INTRODUCTION

Two regions of the human immunodeficiency virus type 1 (HIV-1) gp120 envelope need to engage with cell surface proteins in order to initiate infection: the CD4 binding site (CD4bs) and the chemokine receptor binding site. The latter consists of regions in the V3 loop and the bridging sheet, which includes the β20 and β21 strands of C4 and the β2 and β3 strands of the V1V2 stem (1–8). Some strains of HIV have evolved to be independent of CD4 usage (9, 10), but virus binding to chemokine receptors is essential for infectivity, as demonstrated by the fact that deletion of the V3 region of gp120 completely abrogates infectivity (11). Indeed, the critical functional role of V3 was first described more than 2 decades ago when it was recognized that specific amino acids in V3 determine viral tropism (12, 13).

While the V3 loop plays this essential role in the infectivity of the virus, it is also the target of antibodies (Abs) that are made by essentially all HIV-infected individuals (14–16) and are easily induced by most candidate HIV vaccines (16–21). When V3 is accessible on the surface of the virion, V3-specific Abs efficiently neutralize the virus; this is exemplified by the cross-clade neutralization demonstrated with many tier 1 and some tier 2 viruses (22, 23). While V3 on the unliganded trimer is accessible to some V3 Abs (24), much of V3 is occluded within the unliganded trimeric envelope spike (25–27). The partial and transient nature of the exposure of V3 epitopes on the trimeric envelope spike clarifies much of the controversial data in the literature concerning the neutralizing activity of V3 Abs. As noted, these Abs have been shown to neutralize tier 1 viruses potently but were thought to neutralize most primary isolates poorly or not at all (22, 28). However, recently, it has been shown that V3 Abs (i) can neutralize tier 2 and 3 viruses if Ab and virus are coincubated for 4 to 24 h (23), (ii) play an *in vivo* role in constraining the native Env trimer to a neutralization-resistant phenotype (29), (iii) correlate with reduced infection of infants born to HIV-infected mothers (30), (iv) correlate with a reduced rate of infection in human vaccinees (31–33), and (v) exert immune pressure, reflected in the sequence of the viruses transmitted to vaccinees in the RV144 human vaccine trial (17, 18).

**FIG 1:**
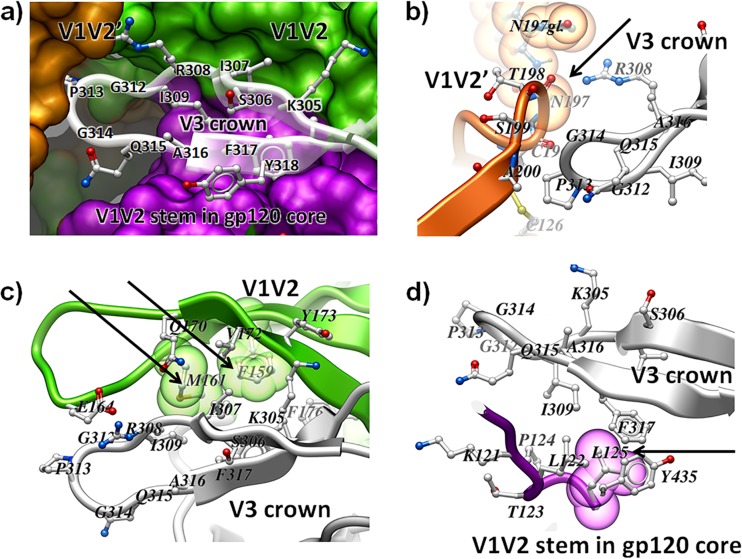
The V3 loop in the context of the gp120 trimer. (a) Overview of the V3 loop environment. The crown of the V3 hairpin (stick representation) is docked in a pocket formed by the V1V2 loop from the same protomer (green), the gp120 core with its V1V2 stem from the same protomer (magenta), as well as the V1V2 stem from the adjacent gp120 protomer (V1V2′; orange). (b to d) Close-ups of the interactions between the V3 crown and other domains. The residues indicated by arrows were mutated in the studies described here and are denoted by space-filling transparent spheres. The structures are reoriented as appropriate to best depict the key features of the respective interfaces. (b) Residue N197 in the β3 strand of the V1V2′ stem (indicated by an arrow) in the adjacent protomer interacts with V3. The first *N*-acetylglucosamine residue of the glycan is also visible and is marked N197gl. Residues F159 and M161 (indicated by arrows) interact with V1V2 of the same protomer (c), and residue L125 in the V1V2 stem in the gp120 core (indicated by an arrow) interacts with the V3 of the same protomer (d). Images were generated from the structure with PDB accession number 4TVP (24).

The V3 loop was originally described to be the principal neutralizing domain (34, 35), but data quickly indicated that the neutralizing potency of V3 Abs was highly dependent on the virus being tested, the epitope specificity of the V3 Ab, and the assay being used (36). The recent literature shows that, for survival of most viral isolates, the V3 loop is protected from the antiviral effects of V3 Abs; however, transition from its partially occluded state to an accessible state occurs as a result of both the conformational plasticity of the envelope and its ligation to CD4. Thus, gp120, like most proteins, is not conformationally fixed but flickers between various energy states even when unbound to ligands; similarly, the V3 region of gp120 flickers between occlusion and accessibility. The conformational dynamics are also characteristic of the envelope trimer on the surface of virions, even when unliganded (37, 38), and many anti-V3 monoclonal antibodies (MAbs) bind to the unliganded SOSIP (24, 39, 40). Additional recent data indicate that V3 functions as part of the trimer-associating domain while residing in a pocket bounded by the V1V2 loop and the V1V2 stem of the gp120 core of the same protomer and by the V1V2 stem of the neighboring protomer (Fig. 1a) (24, 41).

Here, we provide a systematic interrogation of several mechanisms by which single amino acid changes in gp120 render viruses sensitive to neutralization, a demonstration that individual mutations in various regions of the envelope trimer result in altered packing of the V3 loop, and data showing that individual mutations in various regions of gp120 activate an open conformation that exposes V3 to the effects of V3 Abs. Thus, we link data on the molecular interactions that release V3 from its pocket in the unliganded trimer on virions with experimental data showing how these structural alterations render viruses exceptionally sensitive to V3 Abs. Only one of the many mutations that hold V3 in its partially occluded state is sufficient to release it. The data reveal that the envelope trimer uses several methods to maintain V3 in a state that is resistant to V3 Abs but that minor disruptions allow the exposure of V3 with subsequent susceptibility to neutralization by V3 Abs.

## MATERIALS AND METHODS

### Envelope clones and site-directed mutagenesis.

The following envelope clones and their corresponding A204E mutant proteins were described previously (42–44): four subtype A clones (Q23ENV.17 [referred to here as Q23.17], MG505.W0M.ENV.H3 [referred to here as MG505.H3], BG505.W6M.ENV.B1 [referred to here as BG505.B1], QF495.23M.ENV.A3 [referred to here as QF495.A3], one subtype C clone (QC406.70M.ENV.F3 [referred to here as QA406.F3]), and one subtype D clone (QA013.70I.ENV.H1 [referred to here as QA103.H1]). Point mutations were introduced into the gp120 region of plasmid pCAGGS_JR-FL.JB gp160 (kindly supplied by John Mascola) and into gp160 of HIV-1_YU2_ using a QuikChange II XL or QuikChange Lightning Multi site-directed mutagenesis kit (Stratagene, La Jolla, CA, USA) according to the manufacturer's instructions. All mutant gp160 constructs were sequenced completely to confirm the correct amino acid change.

### Pseudovirus production.

The clade A, C, and D pseudoviruses were generated by cotransfecting 293T cells with 0.5 μg of each envelope clone with 1.0 μg of an *env*-deficient subtype A proviral plasmid (Q23Δenv) (45). 293T cells (4 × 10^4^) were plated into the wells of a six-well dish at ∼24 h prior to transfection in complete Dulbecco modified Eagle medium (DMEM). For each transfection, plasmid DNA was mixed with 6 μl of the Fugene 6 transfection reagent (Roche). Pseudoviruses were harvested at 48 h posttransfection. Supernatants were spun at 1,200 × *g* for 5 min at room temperature to remove the cell debris, and aliquots of the pseudovirus stocks were stored at −80°C. The viral titer of each transfection supernatant was determined by infecting TZM.bl cells and counting the number of blue cells at 48 h postinfection after staining for β-galactosidase activity (46). For the JR-FL.JB (referred to here as JR-FL) wild type (WT) and mutants, the pCAGGS_JR-FL.JB plasmid was used to cotransfect 293T cells with a Δ*env* backbone plasmid, pSG3Δenv (from the NIH AIDS Research and Reference Reagent Program), at optimal backbone/envelope ratios, usually 3:1, using the Fugene HD reagent according to the manufacturer's protocol. After 48 to 72 h, supernatants containing the secreted mutant or WT pseudoviruses were harvested, filtered, aliquoted, and stored at −80°C. All pseudoviruses were titrated prior to use (47).

### Neutralization assays.

Neutralization was measured as a function of the reduction in luciferase or β-galactosidase activity after a single round of virus infection in TZM.bl cells, as described previously (47). TZM.bl cells were obtained through the NIH AIDS Research and Reference Reagent Program. Briefly, either ∼500 infectious pseudoviral particles in 25 μl of complete DMEM or the equivalent of 100 to 200 50% tissue culture infective doses (TCID_50_) of each pseudovirus were incubated with 10-fold serial dilutions of each MAb or CD4IgG2 (Progenics Pharmaceuticals, Inc., Tarrytown, NY) for 60 min at 37°C. A total of 1 × 10^4^ TZM.bl cells in 100 μl of complete DMEM were added to each dilution in the presence of DEAE-dextran at a final concentration of 10 to 12 μg/ml. Each MAb or CD4IgG2 was tested at a starting concentration of 50 μg/ml. At 48 h postinfection, neutralization was measured by determination of either β-galactosidase activity with a Galacto-Lite system (Applied Biosystems) or luminescence with Bright-Glo substrate solution (Promega, Madison, WI). The 50% inhibitory concentration (IC_50_) was determined to be the concentration of MAb or CD4IgG2 at which 50% of the pseudovirus input was neutralized, as previously described (46). The IC_50_s reported represent the averages from at least two independent experiments, each of which was performed in triplicate.

### Human monoclonal antibodies.

The glycan-independent V3 MAbs specific for the crown of the V3 loop that were used in this study included 10-188 (48), 1-79 (48), 447 (49), 2219 (50), 2557 (51), 3074 (52), 3869 (53), and 2424 (51). The breadth and potency of neutralization by these MAbs have been published previously (22, 23, 52). The negative-control human MAb used, MAb 1418, is specific for parvovirus B19 (54), and the positive-control fusion protein used was CD4IgG2.

### Flexible loop simulations.

Simulations of the V3 stem-crown and of the B-C strand-connecting loop in the V1V2 domain were performed in Internal Coordinate Mechanics (ICM) (Molsoft, San Diego, CA). The X-ray structure of the cleaved, stabilized, soluble Env trimer (termed BG505 SOSIP.664 gp140) in complex with a potent broadly neutralizing antibody, PGT122 (PDB accession number 4TVP), was used as a starting point (24).

For the V3 stem-crown region, residues 301 through 326 were allowed to flex during Monte Carlo sampling (2 × 10^7^ energy evaluations). Five hundred lowest-energy conformations were derived from 300 simulations with and without the Man9 glycan attached to N301. Conformational stacks (ensembles) were accumulated with a vicinity parameter of 20 degrees (i.e., with representative lowest-energy conformers separated by at least a 20-degree angular root mean square deviation [RMSD] for backbone phi/psi angles) and combined across all runs. The resulting ensemble was used to derive a distribution (a histogram with 3-Å bins) of backbone RMSDs from the original in-pocket conformation of V3 observed in the trimer X-ray structure. The standard error of bin frequencies was estimated according to Poisson statistics, i.e., as a square root of frequency.

For the B-C strand-connecting loop in the V1V2 domain, residues ∼160 through 171 (the region between the C and N termini of the B and C strands, respectively) were allowed to flex during Monte Carlo sampling (10^6^ energy evaluations). Simulations were performed with and without the glycan attached to N160. For the simulations with the full Man9 glycan, a structure in which only the first two *N*-acetylglucosamine monosaccharides of the glycan chain are resolved in the experimental structure was built. The 10 lowest-energy conformations generated with and without N160 glycan were further analyzed.

## RESULTS

Recent crystallographic data for the trimeric Env ectodomain showed that the V3 loop is docked in a pocket formed by the V1V2 domain of its own protomer (intraprotomer interaction), the V1V2 stem in the gp120 core of its own protomer (intraprotomer interaction), and the stem of V1V2 in the adjacent gp120 protomer (interprotomer interaction) (24) (Fig. 1a). In the trimer, the V3 crown occurs in a conformation similar to that in which it is bound by V3-specific MAbs, in which the V3 loop is held in the Ab-combining site like that of an infant in a cradle (55, 56). Destabilization of the V3 interactions with the pocket or other structural changes that disrupt the pocket could release the V3 loop, making it accessible to binding by V3 Abs.

**FIG 2:**
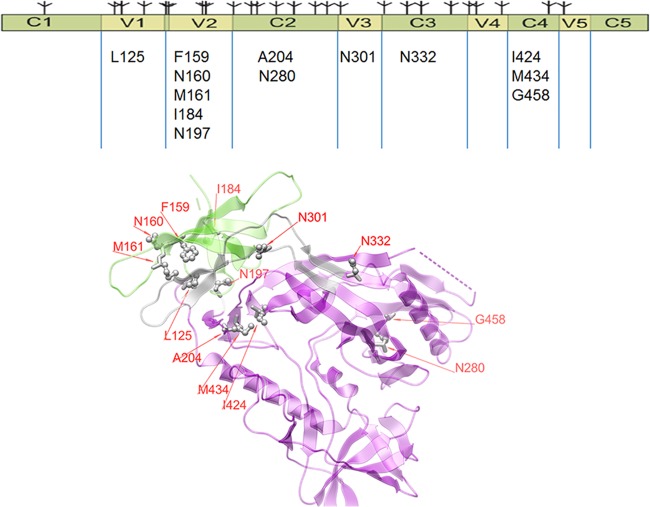
Overview of mutations made in gp120 to assess the impact on exposure of V3. (Top) Linear diagram (90) showing the five constant regions (C1 to C5) and five variable regions (V1 to V5) of gp120 with the amino acids in each region that were mutated individually in this study. Putative glycosylation sites are shown by the symbols above the bar. (Bottom) A two-dimensional depiction of gp120 with mutated residues shown by stick representation. As negative controls, pseudoviruses carrying the V2 mutation V182Q and the C4 mutation N448Q or T450A were constructed but are not shown here. The image was generated from the structure with PDB accession number 4TVP (24).

To investigate the underlying mechanisms by which V3 is held in and released from its pocket, experiments in which single amino acid mutations were made in gp120 of several WT pseudoviruses were designed. Mutations were selected to elucidate the various interactions that hold V3 in its pocket and the perturbations that release it. An overview of the mutations made and their positions in gp120 are shown in Fig. 2.

### Disruption of quaternary interprotomer interactions.

**FIG 3:**
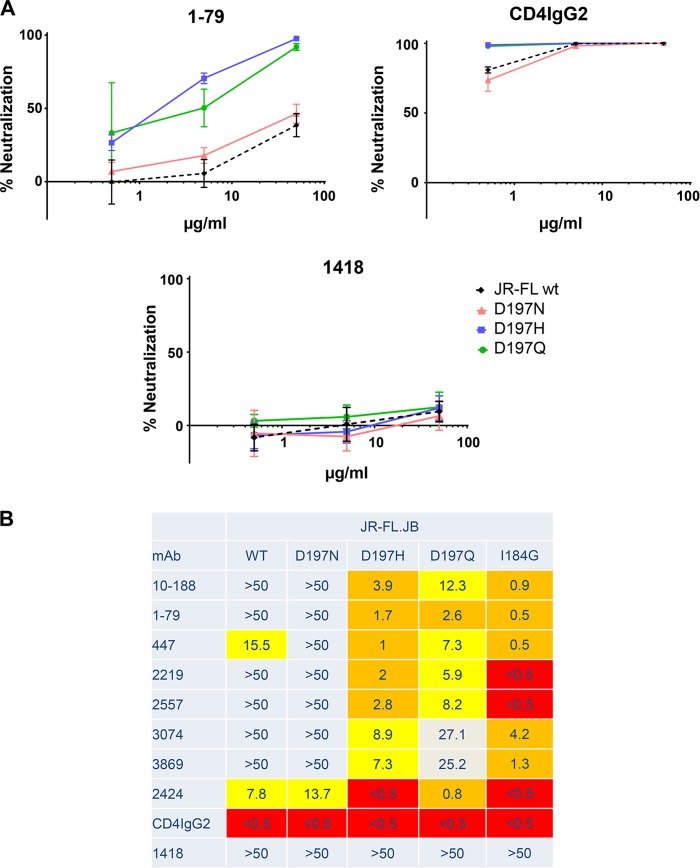
Effects of mutations on neutralization sensitivity of pseudoviruses carrying the WT gp120 of clade B strain JR-FL.JB or Env with mutations at position 197 or 184. (A) Neutralization of JR-FL.JB WT and three mutants with mutations at position 197 by anti-V3 MAb 1-79, soluble CD4IgG2, and antiparvovirus MAb 1418. Dotted lines, neutralization of the WT pseudovirus; solid lines, neutralization of the JR-FL mutants. (B) IC_50_ neutralization values (in micrograms per milliliter) for anti-V3 MAbs against pseudoviruses carrying the envelope of WT clade B strain JR-FL or JR-FL mutants with substitutions at position 197 or 184. Pseudoviruses were also tested with positive and negative controls (CD4IgG2 and MAb 1418, respectively). The TZM.bl cell neutralization assay was used as described above; all experiments were performed in triplicate, and data are shown as the average of all data points from two experiments. Color coding uses red for values of <0.5 μg/ml, orange for values of 0.5 to 5.0 μg/ml, yellow for values of 5.1 to 25 μg/ml, and gray for values of 25 to >50 μg/ml.

Various studies have shown that different regions of the gp120 protomers in the Env spike contribute to the stability of the trimeric form (24–26, 40). For example, Fig. 1c and d show intraprotomer interactions, e.g., interactions of V1V2 and V3 from the same protomer, and Fig. 1b shows interprotomer quaternary interactions, e.g., the interaction of the V1V2 stem from one protomer with the V3 of its neighboring protomer. In order to determine the effect of these interactions on the conformation of V3, we first examined the interactions between the V3 tip and the V1V2 stem in the neighboring protomer (Fig. 1b). This interface on the V1V2 side mostly involves backbone or highly conserved side chain atoms, but position 197 is variable. Previous studies suggested that replacement of the asparagine at 197 with glutamine (N197Q) in the lab-adapted, dual-tropic strain 89.6 removes a putative glycan motif often found at this position in the β3 strand of the V1V2 stem; this resulted in the increased sensitivity of the mutant pseudovirus to V3 MAb 447 (57). Subsequent studies showed that mutation of primary isolates that carry a glycan motif [NX(S/T)] at positions 197 to 199 by replacing the amino acid at position 197 or 199, i.e., N197H or S199P, resulted in increased sensitivity to CD4IgG, heterologous pooled plasma, and MAbs targeting the CD4bs, CD4i, V2, the quaternary neutralizing epitope of PG9, and the MPER epitope in gp41 (58–61). Notably, however, JR-FL has an aspartate at position 197 (D197), abrogating the glycan motif. Given the relative resistance of JR-FL to V3 MAbs (62), it did not appear that the glycan at this position played a role in the occlusion of the V3 loop. To test this, a JR-FL D197N mutant was made to restore the putative glycosylation site; the infectivities of the D197N mutant and the WT were comparable (27,940 TCID_50_/ml). The presence of aspartate or asparagine at position 197 made no difference in neutralization sensitivity (Fig. 3), suggesting that the role played by residue 197 in changing the sensitivity of JR-FL to V3 Abs involves the interaction of amino acids rather than the glycan. In contrast, for the D197H mutant (TCID_50_ = 27,940/ml), the sensitivity to V3 MAbs increased by more than an order of magnitude. Similar results were achieved with the D197Q mutant (TCID_50_ = 12,500/ml), supporting the hypothesis that residue 197 in the V2 loop interacts with the V3 loop from the neighboring gp120 protomer to stabilize it in the pocket that partially occludes its epitopes (Fig. 1b). Similarly, a profound increase in neutralization sensitivity was caused when the I184G mutation was made and tested (Fig. 3B). The isoleucine at position 184 appears to contribute to the quaternary interaction of the V1V2 regions of neighboring protomers, suggesting again that quaternary interprotomer interactions contribute to the positioning of the V3 loop.

### Disruption of the hydrophobic core of the bridging sheet.

**FIG 4:**
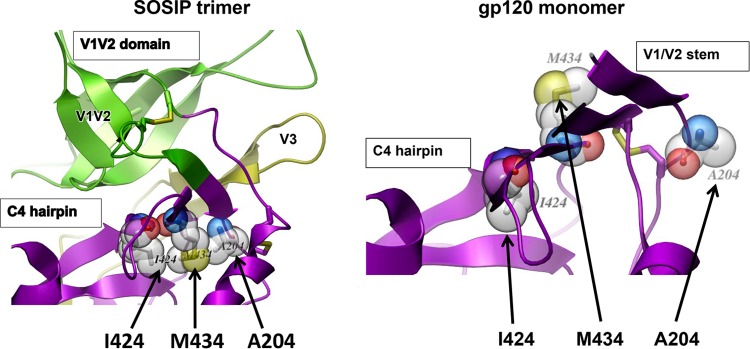
The bridging sheet is an antiparallel β sheet in the structure of the monomer but has a mixed parallel/antiparallel conformation in the SOSIP trimer. (Left) The V2 stem residue A204 and C4 residues M434 and I424 form a buried hydrophobic core in the mixed β sheet of the SOSIP trimer (24). (Right) In contrast, in the structure of the gp120 monomer, there is an antiparallel β-sheet conformation and the three residues (A204, M434, and I424) separate, and two of the side chains become exposed (modified from the structure with PDB accession number 4TVP) (24). Thus, disruption of the trimer core would be expected to alter the packing of the V1V2 stem and/or shift the equilibrium toward the antiparallel configuration of the monomer gp120 bridging sheet structures, with downstream effects on the V1 and V2 loops as well as their interaction with V3.

The bridging sheet of gp120 consists of four β strands: the N-terminal β strand of V1 (β2) and the C-terminal β strand of V2 (β3), the V1V2 stem, and the β20 and β21 strands of C4. The bridging sheet is an antiparallel β sheet in the monomer but assumes a mixed parallel/antiparallel conformation in the SOSIP trimer (63). As shown in Fig. 4, the V2 stem residue A204 and the C4 residues M424 and I434 form a buried hydrophobic core in the mixed β sheet of the SOSIP trimer (24). Notably, in the antiparallel β-sheet conformation in the monomer, the three residues separate and two of the side chains become exposed (Fig. 4). Thus, disruption of this core would be expected to alter the packing of the V1V2 stem and/or to shift the equilibrium toward the antiparallel configuration of the bridging sheet observed in the monomeric gp120 structure, with downstream effects on the V1 and V2 loops as well as their interaction with V3. Indeed, we had previously reported that an I424M mutation found in a clone of a patient isolate rendered the virus highly sensitive to V3 MAbs, and insertion of this mutation into JR-FL, YU2, and RHPA4259.7 also increased the neutralization sensitivity to V3 MAbs (64). In addition, an A204E mutation was shown to affect recognition by MAbs PG9, PG16, and PGT145, which recognize the V2 quaternary epitope (44).

To determine the effects of mutation of the other residues of the bridging sheet hydrophobic core on the sensitivity to V3 Abs, we tested the A204E and M434G mutations in the context of several pseudoviruses. The results, shown in Table 1, indicate the profoundly increased sensitivity of the mutants of the several viruses tested. Both the A204E and M434G mutations increased the sensitivity of JF-FL to all eight V3 MAbs tested by more than 2 orders of magnitude. Six additional pseudoviruses which carried envelopes from clade A, C, or D were tested; each of these WT pseudoviruses were resistant to the eight V3 MAbs when tested at a maximum concentration of 50 μg/ml. In contrast, when the latter viruses with the A204E mutation were tested, five of six were rendered sensitive to two or more V3 MAbs, with the IC_50_s ranging from 0.0005 to 16.0 μg/ml. Notably, the two viruses that remained resistant to most V3 MAbs had unusual mutations in V3 (Table 2), which may have altered the epitopes at the crown of V3 which these V3 MAbs recognize.

**TABLE 1:**
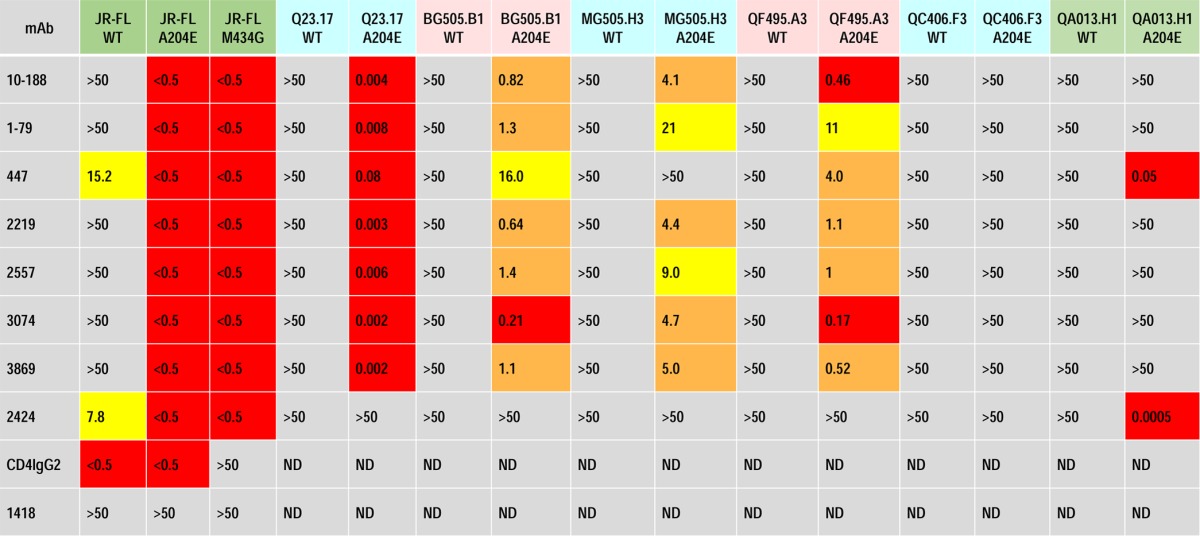
Effects of the A204E mutation in the V2 stem and the M434G mutation in C4 on neutralization sensitivity^*a*^

a IC_50_ neutralization values (in micrograms per milliliter) are shown for pseudoviruses carrying the Envs of several isolates. Pseudoviruses of the WT or with the A204E or M434G mutation were tested with human anti-V3 MAbs and positive and negative controls (CD4IgG2 and antiparvovirus MAb 1418, respectively). The TZM.bl cell neutralization assay was performed in triplicate, and the data are shown as the averages of all data points from two experiments. The following viruses were from the indicated clades: JR-FL, clade B; Q23.17, clade A; BG505.B1, clade A; MG505.H3, clade A; QF495.A3, clade A; QC406.F3, clade C; QA013.H1, clade D. The color coding is described in the legend to Fig. 3.

**TABLE 2:** V3 sequences in wild-type virus isolates that became sensitive or remained resistant to V3 MAbs upon A204E mutation^*a*^

Virus	V3 sequence^*b*^
JR-FL	CTRPNNNTRKSIHIGPGRAFYTTGEIIGDIRQAHC
Q23.17	CIRPNNNTRKSIRIGPGQAFYATGDIIGDIRQAHC
QC406.F3	CTRPNNNTRESI**G**IGPGQ**M**FYAMGAIIGDIRQAHC
QA013.H1	CTRPYNNTRKG**E**HMGPGRALFT**E**R**-**IVGDIRQAYC

a The sensitivity or resistance to V3 MAbs upon A204E mutation is shown in Table 1. JR-FL and Q23.17 were the wild-type virus isolates that became sensitive to V3 MAbs, and QC406.F3 and QA013.H1 were the wild-type virus isolates that remained resistant to V3 MAbs.

b Underlined and bold residues are unusual substitutions within the V3 crown.

### Disruption of intraprotomer interactions of the V3 crown with adjacent regions of gp120.

The V3 crown forms extensive, primarily hydrophobic interactions with adjacent domains within each protomer. Examples are shown in Fig. 1c and d. To examine the intraprotomer interaction between the V3 crown and the loop connecting the B and C strands of V2, mutations were made at position 159 or 161 to disrupt the hydrophobic packing of V2 and V3. Figure 1c shows the interactions of the amino acids at these positions (F159 and M161) in the BG505.B1 infant-derived transmitted variant (46). The F159S and M161Q mutants were each constructed in JR-FL. The effect of these individual mutations on the neutralization sensitivity to V3 MAbs was profound, increasing the sensitivity of all mutants by more than 2 orders of magnitude (Table 3). The hydrophobic face of V3 also interacts with the N-terminal strand of the V1V2 stem in the gp120 core of its own protomer (Fig. 1a and d). To determine if disruption of V3 interactions in this region would liberate V3, an L125F mutation in the V1V2 stem, which we expected would create a steric clash with the V3 F317 side chain, was made (Fig. 1d). The L125F mutation resulted in an increase in sensitivity to V3 MAbs by more than 2 orders of magnitude (Table 3).

**TABLE 3:**
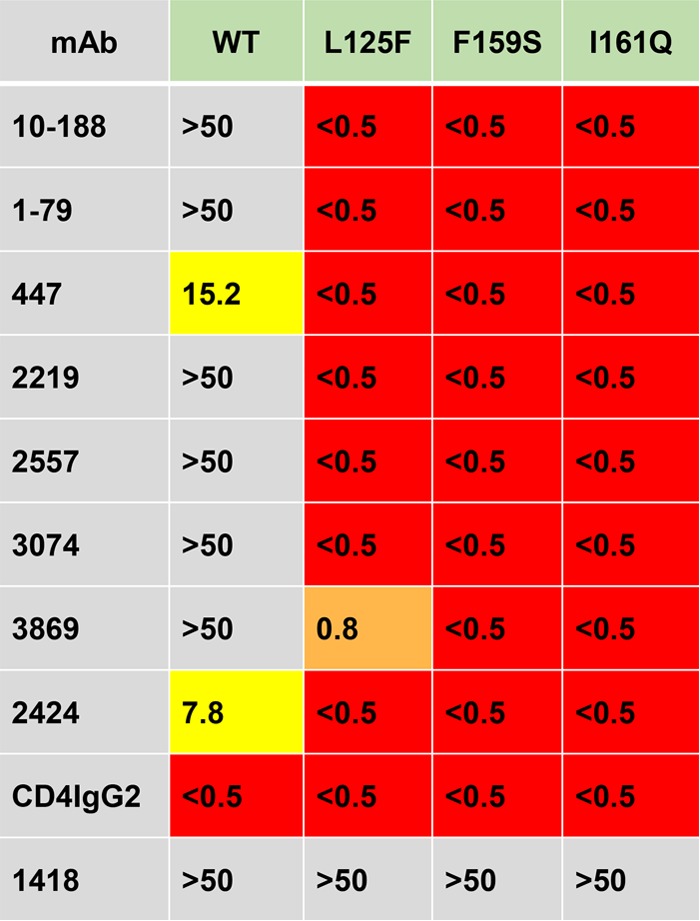
Effects of mutations in JR-FL.JB that disrupt the intraprotomer hydrophobic interactions of V3 with adjacent regions of gp120^*a*^

a The hydrophobic interactions of V3 with adjacent regions of gp120 are shown in Fig. 1c and d. The color coding is described in the legend to Fig. 3.

### Stabilizing role of glycans.

The mobility of flexible regions of a glycoprotein may be affected by nearby glycans. We hypothesized that glycans located close to the base of a flexible loop region could restrict the accessible conformational space of the loop because (i) the bulk of a glycan chain sterically excludes a significant portion of the room that otherwise would be available for loop motions and (ii) the requirement for the outward orientation (i.e., an orientation away from the protein body) of the glycan chain at the point of attachment to the polypeptide locally may restrict loop flexibility since conformations that would make the glycan point toward the protein become prohibited. To test this hypothesis, we performed flexibility simulations for the stem-crown region of V3 and the loop connecting the B and C strands in V2 using as a starting point the X-ray structure of the cleaved, stabilized, soluble Env trimer (BG505 SOSIP.664 gp140) (PDB accession number 4TVP) (24).

**FIG 5:**
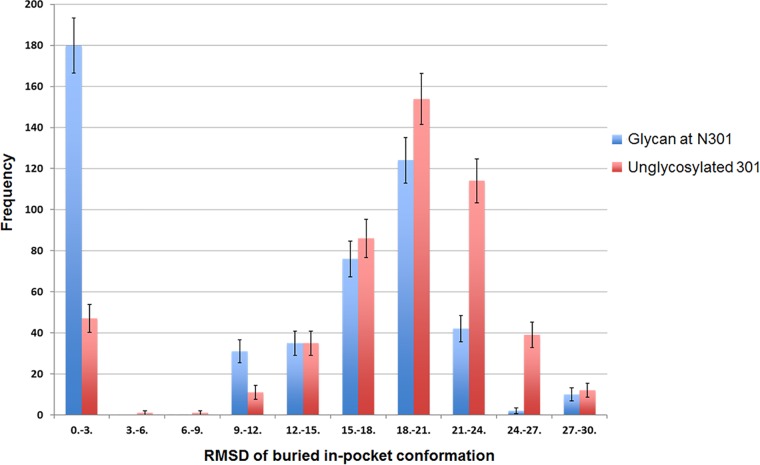
The glycan at N301 restricts the mobility of the V3 loop, sharply increasing the buried conformation. A histogram of RMSDs from the in-pocket state for 500 lowest-energy conformations which was generated by 300 flexibility simulations of the V3 stem and crown in the presence (blue bars) or absence (red bars) of the glycan at position 301 is shown. RMSDs near zero correspond to the V3 buried (in-pocket) conformation, while high RMSDs indicate the released state. Estimated error bars represent the square root of the frequencies (Poisson statistics).

**FIG 6:**
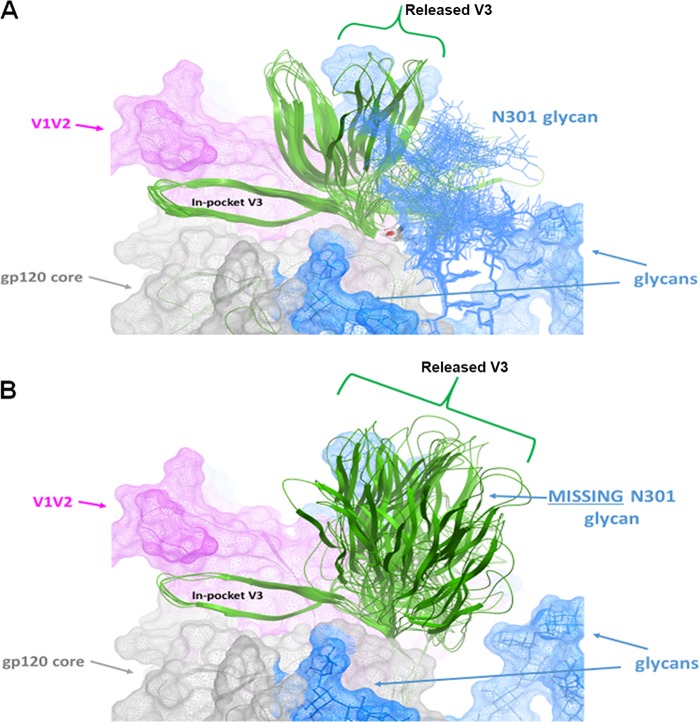
Simulations of the lowest-energy conformations of V3 in the presence and absence of the Man9 glycan attached to N301. The proportions of released and in-pocket conformations of the V3 loops are shown for the 50 lowest-energy V3 conformations (100 Monte Carlo simulations) in the presence of the glycan at N301 (A) and for the 50 lowest-energy V3 conformations (100 Monte Carlo simulations) in the absence of the glycan at N301 (B).

Ensembles of generated V3 conformations were analyzed to determine the populations of exposed and buried states in the presence or absence of the glycan at position N301. As seen in Fig. 5, while the majority of low-energy conformations for the glycosylated loop (blue bars) had the V3 crown buried (low RMSDs), removal of the N301 glycan (red bars) resulted in a sharp increase in the released configuration of V3 (high RMSDs). This can be observed visually in Fig. 6, where the proportion of released V3 loops is much larger in the absence of the glycan at N301 (at the N-terminal end of V3) than in its presence. It is also noteworthy that, even in some of the released conformations, the V3 crown is wedged between N301 and N156 glycans (Fig. 6A) when the N301 glycan is present and therefore is still poorly accessible. The modeling data are supported by the experimental data showing that when N301 was mutated to either aspartate or tyrosine (N301D or N301Y; Table 4), large increases in sensitivity to V3 MAbs (greater than 1 order of magnitude) occurred, indicating that the V3 loop became more frequently available for Ab binding.

**TABLE 4:**
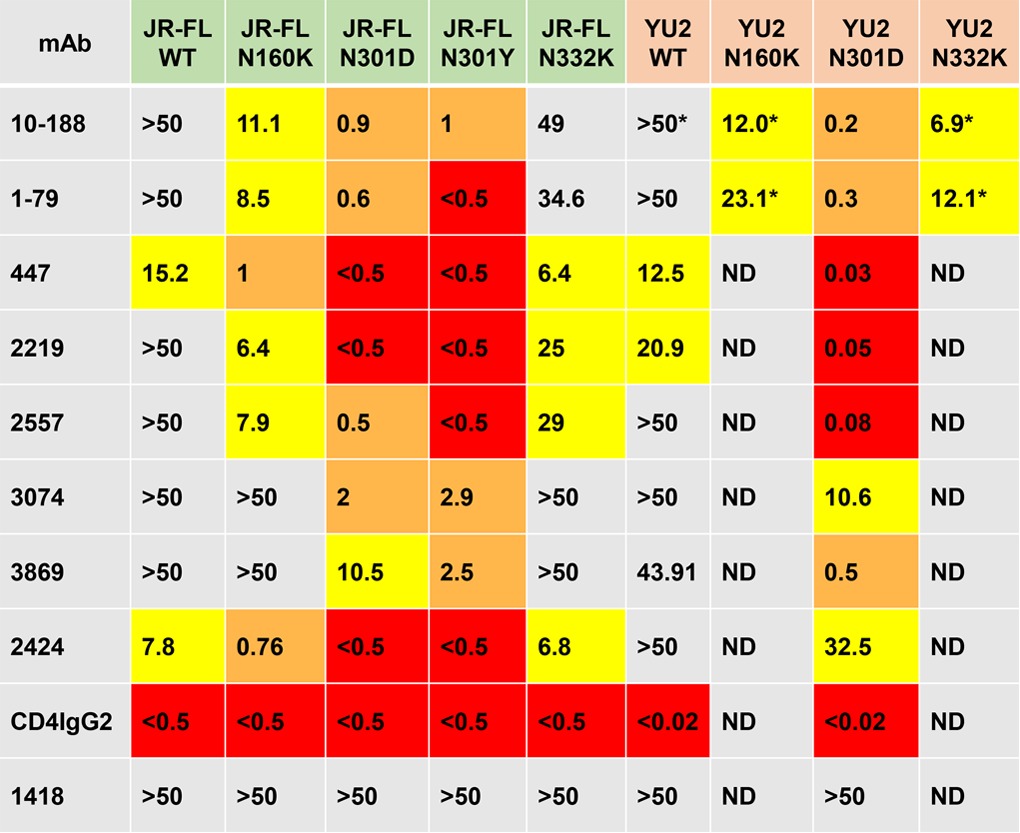
Effects of glycan mutations on neutralization sensitivity^*a*^

a IC_50_ neutralization values (in micrograms per milliliter) are shown for JR-FL and YU2 pseudoviruses carrying glycan mutations. Pseudoviruses were tested with human anti-V3 MAbs. CD4IgG2 was used as a positive neutralization control, and human MAb 1418, specific for parvovirus, was used as a negative control. The TZM.bl cell neutralization assay was performed in triplicate with the JR-FL WT and mutants, and data are shown as the average of all data points from two experiments. The YU2 WT and mutants were tested as previously described (89). The color coding is described in the legend to Fig. 3. ND, not done; *, data from Klein et al. (48).

**FIG 7:**
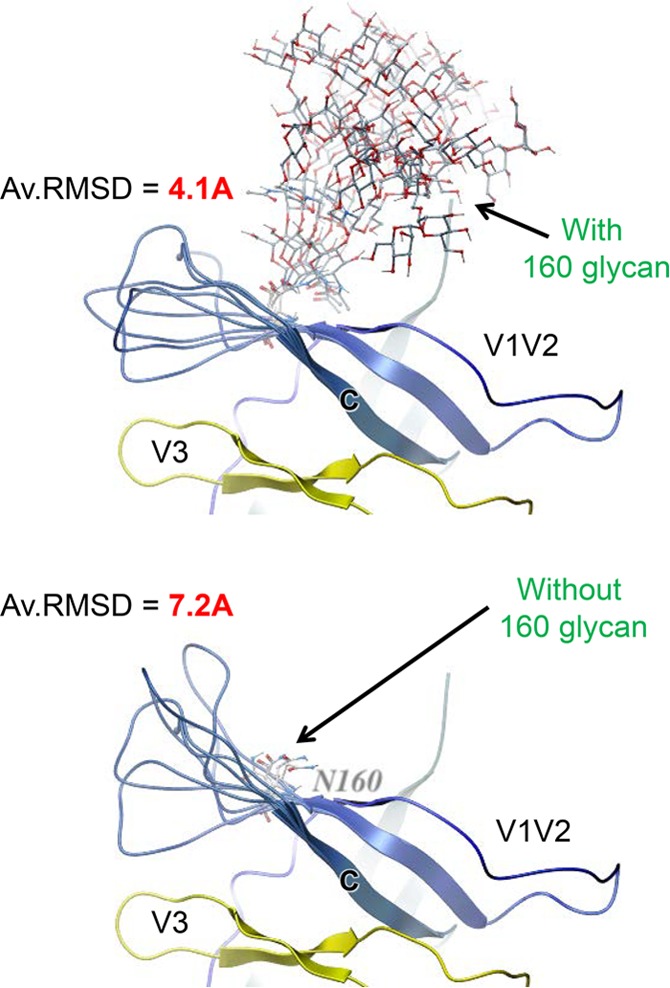
The glycan at N160 can stabilize the flexible elements of V2. Representative conformations from flexibility simulations of the loop that connects the B and C strands of V2 in the presence (upper) and absence (lower) of the glycan at position 160 are shown. The glycan reduces the mobility of the flexible loop.

Similarly, when we performed a simulation of conformational fluctuations of the loop connecting the B and C β strands of V2 (residues 160 to 171 in HxB2) with and without the glycan at N160, we found that the amplitude of loop movements became significantly larger when the N160 glycan was removed, as shown in Fig. 7. In the presence of the N160 glycan (at the N-terminal end of V2), the connecting loop has an average RMSD of 4.1 Å, whereas in the absence of the N160 glycan, the average RMSD of this B-C strand-connecting loop is much larger, 7.2 Å. The greater flexibility of the V2 B-C strand-connecting loop in the absence of N160 would appear to destabilize the V3 binding pocket. When N160K mutants were made, the TCID_50_ of the JR.FL N160K mutant was unchanged from that of the WT (27,970/ml for both), but Table 4 shows that the N160K mutants of JR-FL and YU2 were frequently more than 1 order of magnitude more sensitive than the WT to most V3 MAbs tested.

Interestingly, although mutation of the glycosylation motif at N332 (at the N-terminal end of C3, depicted in Fig. 6) increased the infectivity dramatically over that of the WT (TCID_50_ = 698,760/ml and 27,940/ml, respectively), this mutation had a much less profound effect on the sensitivity of JR-FL and YU2 to V3 MAbs than glycan mutations at positions 160 and 301: there was either no effect of the N332K mutations on the sensitivity to most V3 MAbs or an increase in sensitivity of only 2-fold (Table 4). These data indicate that the glycans at positions 160 and 301 stabilize the highly flexible crown and stem of V3 (roughly between residues 300 and 328), thus affecting the packing of V2 and V3. In contrast, residue N332 is part of the secondary and tertiary structures of the outer domain, contributing to the rather rigid jelly roll structure formed by the residues at the N- and C-terminal V3 base. Therefore, while possibly affecting the global gp120 conformation, this glycan does not significantly affect the space that is potentially accessible for local V3 flexing.

### Disruption of the CD4 binding site.

**FIG 8:**
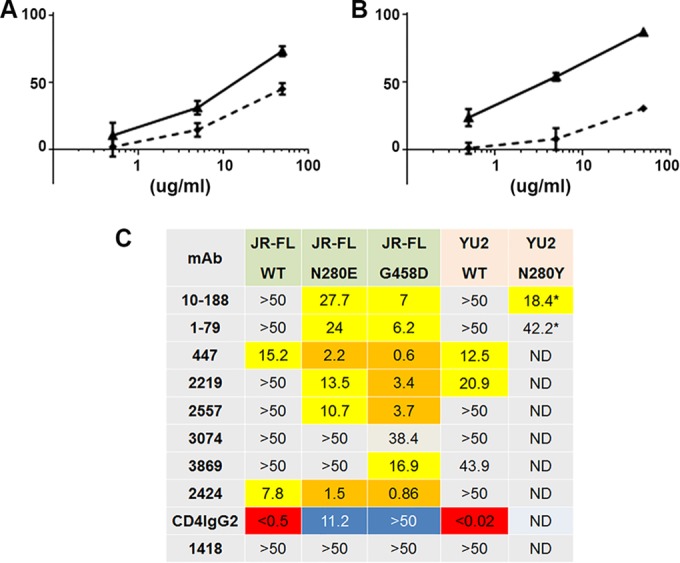
Effect of mutations in the CD4 binding site on neutralization sensitivity. (A) Neutralization sensitivity of the JR-FL N280E mutant to V3 MAb 2219. Solid line, the JR-FL N280E mutant; dashed line, the JR-FL wild type. *x* axis, % neutrallization. (B) Neutralization of the JR-FL G458D mutant to V3 MAb 2219. The lines are as described in the legend to panel A. *x* axis, % neutralization. (C) IC_50_ neutralization values for JR-FL and YU2 WT and mutant pseudoviruses tested with human anti-V3 MAbs. The colors are as described in the legend to Fig. 3 but with the addition of blue boxes, which denote increased resistance to neutralization by CD4IgG2. ND, not done; *, data from reference 48.

Escape mutants have been generated by treating HIV-infected humanized mice and simian-human immunodeficiency virus (SHIV)-infected macaques with MAbs that target the CD4bs (48, 65). Two mutations that have been identified in these studies occur at residues 280 and 458, positions known to participate in the binding of gp120 to CD4 (65). To determine how mutations in the CD4bs affect the exposure of V3, mutations at these sites were made in JR-FL and YU2. The results, shown in Fig. 8, indicate that the N280E (in C2 of JR-FL) and N280Y (in YU2) mutations had minimal effects, increasing the sensitivity to V3 MAbs only marginally. The C4 G458D mutation in JR-FL resulted in a somewhat greater effect on V3 MAb sensitivity, but mutations at none of these sites produced the profound increase in sensitivity to V3 MAbs that were induced by the mutations at the other sites described above. Interestingly, the TCID_50_ of the JR-FL 280E mutant was 139,760/ml and that of the G458D mutant was 2,500/ml, whereas that of the WT was 27,940/ml; despite the quite different effects of these mutations on infectivity, the neutralization of these mutants by anti-V3 MAbs was relatively unaffected.

As additional controls, pseudoviruses carrying the C4 mutation N448Q or T450A or the V2 mutation V182Q were constructed. The C4 mutations were chosen because they shield a T cell epitope and were not expected to affect the exposure of V3 (66), and V182Q was selected because position 182 is an exposed position not involved in any interdomain or quaternary interactions. The sensitivities of the mutants with these mutations to all of the V3 MAbs tested as well as CD4IgG and the control MAb 1418 were unchanged from the sensitivity of the JR-FL WT (data not shown).

## DISCUSSION

Several previous studies have identified naturally occurring isoforms of gp120 in HIV variants circulating *in vivo* that render these viruses extremely susceptible to V3-specific and other Abs (64, 67–71). Many sites have also been identified by mutating pseudoviruses so that their neutralization sensitivity is increased over that of their parental WT viruses (59, 60, 72–75). However, there has been no systematic interrogation of the mechanism(s) by which single amino acid changes in various regions of gp120 render tier 2 viruses more sensitive to neutralization by Abs whose activity is primarily observed with tier 1 viruses. Elucidation of this phenomenon takes on heightened importance, given recent studies showing that V3 Abs exert immune pressure on circulating and transmitted viruses and may play a role in reducing the rate of infection in adults and in infants born to infected mothers (17, 18, 29, 30). Here, we show that individual mutations in various regions of the envelope trimer result in altered packing of the V3 loop, exposing epitopes on V3 and rendering viruses highly sensitive to V3 Abs. These data provide both structural and functional evidence that V3 exists in the unliganded trimer in a metastable state and that individual mutations in various regions of gp120 result in an activated open conformation in which V3 is fully accessible to the effects of V3 Abs.

The trimeric envelope spike on the surface of HIV virions is characterized by structural plasticity even in the absence of bound ligands; thus, the unliganded trimer flickers between closed, intermediate, and open conformations (23, 76–78), although the closed ground state is energetically favored (37, 38). In the closed ground state conformation of the prefusion envelope, many (but not all) of the epitopes targeted by V3-specific Abs are unavailable due to conformational masking (27, 39, 79). Binding to CD4 results in profound structural changes; stabilizes the activated open configuration, exposing V3; and renders it susceptible to the functional properties of V3 Abs (27, 37, 38, 78).

Here we identify four major mechanisms that contribute to the packing of V3 into its metastable pocket in the unliganded trimer: (i) quaternary interprotomer interactions, (ii) hydrophobic packing of the core of the bridging sheet that maintains the V1V2 domain in a position necessary for the formation of the V3 binding pocket, (iii) intraprotomer interactions of the V3 crown with adjacent regions of gp120, and (iv) limited flexibility of the V2 region mediated by the presence of selected glycans. Disruption of any one of these interactions by mutation of selected single amino acids leads to dramatic conformational changes resulting in the exposure of the V3 loop and enhanced sensitivity to neutralization by V3 Abs. This may explain why certain residues in gp120 are strictly conserved in order to hold V3 in its gp120 pocket. For example, N301 and A204 are present in >97% of isolates (80; http://www.hiv.lanl.gov/content/sequence/HIV/mainpage.html). Interestingly, several lab-adapted strains have substitutions of the conserved amino acids at positions shown to expose V3, which may explain the sensitivity of lab-adapted strains to V3 Abs. For example, strain RF has V424, MN has T332 (and no glycan at 334), and HxB2 has no glycan at position 149, consistent with the data presented herein and published by others showing that viruses with mutations from the conserved sequences at these positions have increased sensitivity to V3 and other Abs (81).

The published data describing the dynamic flexibility of the unliganded ground state of the trimer provide an explanation for the function of Abs directed against V3 which do not neutralize most primary isolates or do so poorly in the standard *in vitro* neutralization assay (47). Thus, V3 Abs can eliminate viruses whose trimers readily expose V3 epitopes. As a result, circulating viruses isolated from patients are rarely sensitive to V3 Abs (82, 83), and the exceptional circulating virus that is V3 Ab sensitive often carries one or more mutations at particular residues that, as shown herein, expose V3 (64, 68, 69). In addition, V3 Abs can reduce HIV infection by and exert immune pressure on circulating viruses. Recently, Permar et al. (30) showed that the magnitude of the maternal IgG response specific for the V3 loop was predictive of a reduced risk of maternal-to-child transmission. Data from the RV144 clinical vaccine trial also suggested that there was Ab-mediated blockade of viruses possessing I307 in V3, a component of an epitope recognized by vaccine-induced V3 Abs (17, 31–33, 84, 85). Moreover, prolonging exposure of virus to anti-V3 Abs from 1 h to 24 h results in enhanced effective neutralization (23), and this finding is consistent with the findings of a study that showed that optimal and maximum exposure of V3 on gp120 after addition of CD4 required ≥24 h (27). Together, these data suggest that conformationally dynamic envelope structures can result in the exposure of V3 epitopes that are normally occluded in the unliganded ground state of the trimer and that infection- and vaccine-induced V3 Abs block infection and eliminate circulating viruses that are sensitive to V3 Abs.

We note that unliganded HIV-1 Env is intrinsically dynamic, transitioning between three distinct global prefusion conformations that are affected by CD4 and Ab binding (27, 37, 38). The modeling described herein was performed on the basis of the crystallographic analysis of the SOSIP gp140 trimer bound to the PGT122 MAb (25), and as such, the model represents only one of the conformations most populated in neutralization-resistant virus isolates. Local disruption of interactions in the closed state may or may not eventually lead to the global shift toward other states. We observed that the infectivity of the mutants in this study, with few exceptions, remained similar to that of the WT, providing some indication that they do not necessarily experience a global shift toward a fully open (and more infectious) trimer conformation but possibly experience a more localized opening around the V3 loop. Future studies probing the mutants with other, non-V3 Abs will help establish how far these conformational changes propagate. In this context, the effects of the glycans at positions 160 and 301 (Fig. 5 to 7 and Table 4), as well as mutations in glycan motifs that often appear at other positions, e.g., N136, N156, N160, N197, and N280, contribute to viral resistance to V3-specific and other Abs (67–69, 72, 81, 86, 87). Crystallographic studies revealed that glycans have a stabilizing role on the gp120 core (87), and here we describe that the mobility of flexible regions of a glycoprotein is affected by nearby glycans (Fig. 5 to 7) (27, 37, 38). However, removal of the glycan at position 160 tended to have less dramatic effects on V3 exposure than changes that affect hydrophobic intra- and interprotomer interactions; this suggests that modification of glycosylation may be a strategy employed by Env to fine-tune exposure of V3 with less dramatic conformational changes as selective pressures change during the course of infection.

It is noteworthy that mutations in the CD4bs had a relatively minor impact on neutralization sensitivity to V3 Abs. In contrast to the effects of the N280E and G458D mutations on CD4 binding, which decreased the neutralizing sensitivity of CD4IgG2 by ≥100-fold, these mutations had only a modest effect on sensitivity to anti-V3 MAbs, not affecting neutralization sensitivity at all or increasing sensitivity by only severalfold (Fig. 8). This is similar to previous findings, where mutation of residue 384, also part of the CD4bs, resulted in only a 6- to 13-fold increase in sensitivity to Abs in HIV-positive sera (58). The effects of these CD4bs mutations on residues buried in the CD4bs are likely to have an indirect effect on V3 MAb neutralization rather than a direct effect on the exposure of V3. One possible scenario suggests that with the decreased affinity for CD4, the conformational changes induced by ligation of CD4 to gp120 may be minimized, thus precluding full exposure of V3.

The use of MAbs for immunotherapy presents a different problem for the virus. Under these conditions, all viruses sensitive to the antiviral effects of the administered MAb will be eliminated; however, selection for mutant species will occur. Many of these selected variants have increased sensitivity to V3 Abs, suggesting that the mutants needed for escape from immunotherapy are forced to adopt envelope configurations that are preferentially in a more open configuration and hence more easily neutralized. For example, studies in YU2-infected humanized mice showed that administration of MAb PG16 resulted in a mutation at position 160 (48). Similarly, nonhuman primates infected with SHIV_AD8EO_ and passively immunized with V3 glycan-specific MAb 10-1074 selected for escape variants with mutations at position 332 (88). Mutations at these positions result in increased sensitivity to V3 Abs, as shown herein. Moreover, when YU2-infected humanized mice were passively immunized with MAb 10-1074 and a V3-specific MAb, 1-79, their viral load decreased by 3 orders of magnitude and remained below the limit of detection with no viral escape during 6 weeks of therapy (48).

In summary, V3 has been shown to exist in a metastable state in which many of its epitopes are occluded by its position in a pocket formed by inter- and intraprotomer interactions with the loop and/or stem of V1V2 (Fig. 1a). However, single mutations in many regions of gp120 release V3 from this pocket, rendering V3 fully accessible to V3 Ab binding and imparting high sensitivity to the neutralizing activity of V3 Abs. These data, taken together with the structural plasticity of gp120 in its unliganded ground state, indicate that V3 can be transiently exposed on the surface of the intact infectious virion and suggest how V3-specific Abs can exert immune pressure on circulating viruses and influence both the nature of transmitted viruses and the rate of infection.

## References

[1] Biscone MJ, Miamidian JL, Muchiri JM, Baik SS, Lee FH, Doms RW, Reeves JD 2006 Functional impact of HIV coreceptor-binding site mutations. Virology 351:226–236. doi:10.1016/j.virol.2006.03.017. 16631222

[2] Choe H, Farzan M, Sun Y, Sullivan N, Rollins B, Ponath PD, Wu L, Mackay CR, LaRosa G, Newman W, Gerard N, Gerard C, Sodroski J 1996 The beta-chemokine receptors CCR3 and CCR5 facilitate infection by primary HIV-1 isolates. Cell 85:1135–1148. doi:10.1016/S0092-8674(00)81313-6. 8674119

[3] Cocchi F, DeVico AL, Garzino-Demo A, Cara A, Gallo RC, Lusso P 1996 The V3 domain of the HIV-1 gp120 envelope glycoprotein is critical for chemokine-mediated blockade of infection. Nat Med 2:1244–1247. doi:10.1038/nm1196-1244. 8898753

[4] Trkola A, Dragic T, Arthos J, Binley JM, Olson WC, Allaway GP, Cheng-Mayer C, Robinson J, Maddon PJ, Moore JP 1996 CD4-dependent, antibody-sensitive interactions between HIV-1 and its co-receptor CCR-5. Nature 384:184–187. doi:10.1038/384184a0. 8906796

[5] Hill CM, Deng HK, Unutmaz D, KewalRamani VN, Bastiani L, Gorny MK, Zolla-Pazner S, Littman DR 1997 Envelope glycoproteins from human immunodeficiency virus types 1 and 2 and simian immunodeficiency virus can use human CCR5 as a coreceptor for viral entry and make direct CD4-dependent interactions with this chemokine receptor. J Virol 71:6296–6304. 9261346 10.1128/jvi.71.9.6296-6304.1997 PMC191902

[6] Hu Q, Napier KB, Trent JO, Wang Z, Taylor S, Griffin GE, Peiper SC, Shattock RJ 2005 Restricted variable residues in the C-terminal segment of HIV-1 V3 loop regulate the molecular anatomy of CCR5 utilization. J Mol Biol 350:699–712. doi:10.1016/j.jmb.2005.05.024. 15964018

[7] Cormier EG, Dragic T 2002 The crown and stem of the V3 loop play distinct roles in human immunodeficiency virus type 1 envelope glycoprotein interactions with the CCR5 coreceptor. J Virol 76:8953–8957. doi:10.1128/JVI.76.17.8953-8957.2002. 12163614 PMC136967

[8] Nolan KM, Jordan AP, Hoxie JA 2008 Effects of partial deletions within the human immunodeficiency virus type 1 V3 loop on coreceptor tropism and sensitivity to entry inhibitors. J Virol 82:664–673. doi:10.1128/JVI.01793-07. 17977968 PMC2224606

[9] Hoffman TL, LaBranche CC, Zhang W, Canziani G, Robinson J, Chaiken I, Hoxie JA, Doms RW 1999 Stable exposure of the coreceptor-binding site in a CD4-independent HIV-1 envelope protein. Proc Natl Acad Sci U S A 96:6359–6364. doi:10.1073/pnas.96.11.6359. 10339592 PMC26886

[10] Edwards TG, Hoffman TL, Baribaud F, Wyss S, LaBranche CC, Romano J, Adkinson J, Sharron M, Hoxie JA, Doms RW 2001 Relationships between CD4 independence, neutralization sensitivity, and exposure of a CD4-induced epitope in a human immunodeficiency virus type 1 envelope protein. J Virol 75:5230–5239. doi:10.1128/JVI.75.11.5230-5239.2001. 11333905 PMC114929

[11] Saunders CJ, McCaffrey RA, Zharkikh I, Kraft Z, Malenbaum SE, Burke B, Cheng-Mayer C, Stamatatos L 2005 The V1, V2, and V3 regions of the human immunodeficiency virus type 1 envelope differentially affect the viral phenotype in an isolate-dependent manner. J Virol 79:9069–9080. doi:10.1128/JVI.79.14.9069-9080.2005. 15994801 PMC1168758

[12] Shioda T, Levy JA, Cheng-Mayer C 1992 Small amino acid changes in the V3 hypervariable region of gp120 can affect the T-cell-line and macrophage tropism of human immunodeficiency virus type 1. Proc Natl Acad Sci U S A 89:9434–9438. doi:10.1073/pnas.89.20.9434. 1409653 PMC50146

[13] Fouchier RA, Brouwer M, Broersen SM, Schuitemaker H 1995 Simple determination of human immunodeficiency virus type 1 syncytium-inducing V3 genotype by PCR. J Clin Microbiol 33:906–911. 7790458 10.1128/jcm.33.4.906-911.1995 PMC228065

[14] Zolla-Pazner S 2005 Improving on nature: focusing the immune response on the V3 loop. Hum Antibodies 14:69–72. 16720976

[15] Tomaras GD, Yates NL, Liu P, Qin L, Fouda GG, Chavez LL, Decamp AC, Parks RJ, Ashley VC, Lucas JT, Cohen M, Eron J, Hicks CB, Liao HX, Self SG, Landucci G, Forthal DN, Weinhold KJ, Keele BF, Hahn BH, Greenberg ML, Morris L, Karim SS, Blattner WA, Montefiori DC, Shaw GM, Perelson AS, Haynes BF 2008 Initial B-cell responses to transmitted human immunodeficiency virus type 1: virion-binding immunoglobulin M (IgM) and IgG antibodies followed by plasma anti-gp41 antibodies with ineffective control of initial viremia. J Virol 82:12449–12463. doi:10.1128/JVI.01708-08. 18842730 PMC2593361

[16] Davis KL, Gray ES, Moore PL, Decker JM, Salomon A, Montefiori DC, Graham BS, Keefer MC, Pinter A, Morris L, Hahn BH, Shaw GM 2009 High titer HIV-1 V3-specific antibodies with broad reactivity but low neutralizing potency in acute infection and following vaccination. Virology 387:414–426. doi:10.1016/j.virol.2009.02.022. 19298995 PMC2792036

[17] Zolla-Pazner S, Edlefsen PT, Rolland M, Kong X-P, DeCamp AC, Gottardo R, Williams C, Tovanabutra S, Sharpe-Cohen S, Mullins JI, DeSouza MS, Karasavvas N, Nitayaphan S, Rerks-Ngarm S, Pitisuttithum P, Kaewkungwal J, O'Connell RJ, Robb ML, Michael NL, Kim JH, Gilbert P 2014 Vaccine-induced human antibodies specific for the third variable region of HIV-1 gp120 impose immune pressure on infecting viruses. eBiomedicine 1:37–45. 25599085 10.1016/j.ebiom.2014.10.022 PMC4293639

[18] Gottardo R, Bailer RT, Korber BT, Gnanakaran S, Phillips J, Shen X, Tomaras GD, Turk E, Imholte G, Eckler L, Wenschuh H, Zerweck J, Greene K, Gao H, Berman PW, Francis D, Sinangil F, Lee C, Nitayaphan S, Rerks-Ngarm S, Kaewkungwal J, Pitisuttithum P, Tartaglia J, Robb ML, Michael NL, Kim JH, Zolla-Pazner S, Haynes BF, Mascola JR, Self S, Gilbert P, Montefiori DC 2013 Plasma IgG to linear epitopes in the V2 and V3 regions of HIV-1 gp120 as correlates of infection risk in the RV144 vaccine efficacy trial. PLoS One 8:e75665. doi:10.1371/journal.pone.0075665. 24086607 PMC3784573

[19] Vaine M, Wang S, Liu Q, Arthos J, Montefiori D, Goepfert P, McElrath MJ, Lu S 2010 Profiles of human serum antibody responses elicited by three leading HIV vaccines focusing on the induction of Env-specific antibodies. PLoS One 5:e13916. doi:10.1371/journal.pone.0013916. 21085486 PMC2976701

[20] Letvin NL, Robinson S, Rohne D, Axthelm MK, Fanton JW, Bilska M, Palker TJ, Liao HX, Haynes BF, Montefiori DC 2001 Vaccine-elicited V3 loop-specific antibodies in rhesus monkeys and control of a simian-human immunodeficiency virus expressing a primary patient human immunodeficiency virus type 1 isolate envelope. J Virol 75:4165–4175. doi:10.1128/JVI.75.9.4165-4175.2001. 11287566 PMC114162

[21] Beddows S, Schulke N, Kirschner M, Barnes K, Franti M, Michael E, Ketas T, Sanders RW, Maddon PJ, Olson WC, Moore JP 2005 Evaluating the immunogenicity of a disulfide-stabilized, cleaved, trimeric form of the envelope glycoprotein complex of human immunodeficiency virus type 1. J Virol 79:8812–8827. doi:10.1128/JVI.79.14.8812-8827.2005. 15994775 PMC1168742

[22] Hioe CE, Wrin T, Seaman MS, Yu X, Wood B, Self S, Williams C, Gorny MK, Zolla-Pazner S 2010 Anti-V3 monoclonal antibodies display broad neutralizing activities against multiple HIV-1 subtypes. PLoS One 5:e10254. doi:10.1371/journal.pone.0010254. 20421997 PMC2858080

[23] Upadhyay C, Mayr LM, Zhang J, Kumar R, Gorny MK, Nadas A, Zolla-Pazner S, Hioe CE 2014 Distinct mechanisms regulate exposure of neutralizing epitopes in the V2 and V3 loops of HIV-1 envelope. J Virol 88:12853–12865. doi:10.1128/JVI.02125-14. 25165106 PMC4248937

[24] Pancera M, Zhou T, Druz A, Georgiev IS, Soto C, Gorman J, Huang J, Acharya P, Chuang G-Y, Ofek G, Stewart-Jones GBE, Stuckey J, Bailer RT, Joyce MG, Louder MK, Tumba N, Yang Y, Zhang B, Cohen MS, Haynes BF, Mascola JR, Morris L, Munro JB, Blanchard SC, Mothes W, Connors M, Kwong PD 2014 Structure and immune recognition of trimeric pre-fusion HIV-1 Env. Nature 514:455–461. doi:10.1038/nature13808. 25296255 PMC4348022

[25] Julien J-P, Cupo A, Sok D, Stanfield RL, Lyumkis D, Deller MC, Klasse P-J, Burton DR, Sanders RW, Moore JP, Ward AB, Wilson IA 2013 Crystal structure of a soluble cleaved HIV-1 envelope trimer. Science 342:1477–1483. doi:10.1126/science.1245625. 24179159 PMC3886632

[26] Lyumkis D, Julien JP, de Val N, Cupo A, Potter CS, Klasse PJ, Burton DR, Sanders RW, Moore JP, Carragher B, Wilson IA, Ward AB 2013 Cryo-EM structure of a fully glycosylated soluble cleaved HIV-1 envelope trimer. Science 342:1484–1490. doi:10.1126/science.1245627. 24179160 PMC3954647

[27] Do Kwon Y, Pancera M, Acharya P, Georgiev IS, Crooks ET, Gorman J, Joyce MG, Guttman M, Ma X, Narpala S, Soto C, Terry DS, Yang Y, Zhou T, Ahlsen G, Bailer RT, Chambers M, Chuang GY, Doria-Rose NA, Druz A, Hallen MA, Harned A, Kirys T, Louder MK, O'Dell S, Ofek G, Osawa K, Prabhakaran M, Sastry M, Stewart-Jones GB, Stuckey J, Thomas PV, Tittley T, Williams C, Zhang B, Zhao H, Zhou Z, Donald BR, Lee LK, Zolla-Pazner S, Baxa U, Schon A, Freire E, Shapiro L, Lee KK, Arthos J, Munro JB, Blanchard SC, Mothes W, Binley JM, McDermott AB, Mascola JR, Kwong PD 2015 Crystal structure, conformational fixation, and receptor interactions of mature unliganded HIV-1 Env. Nat Struct Mol Biol 22:522–531. doi:10.1038/nsmb.3051. 26098315 PMC4706170

[28] Binley JM, Wrin T, Korber B, Zwick MB, Wang M, Chappey C, Stiegler G, Kunert R, Zolla-Pazner S, Katinger H, Petropoulos CJ, Burton DR 2004 Comprehensive cross-clade neutralization analysis of a panel of anti-human immunodeficiency virus type 1 monoclonal antibodies. J Virol 78:13232–13252. doi:10.1128/JVI.78.23.13232-13252.2004. 15542675 PMC524984

[29] Moody MA, Gao F, Gurley TC, Amos JD, Kumar A, Hora B, Marshall DJ, Whitesides JF, Xia S-M, Parks RJ, Lloyd KE, Hwang KK, Lu X, Bonsignori M, Finzi A, Vandergrift NA, Alam SM, Ferrari G, Shen X, Tomaras G, Kamanga G, Cohen MS, Sam N, Kapiga S, Gray ES, Tumba NL, Morris L, Zolla-Pazner S, Gorny MK, Mascola JR, Hahn BH, Shaw GM, Sodroski J, Liao HX, Montefiori D, Hraber P, Korber B, Haynes BF 2015 Common envelope V3 and CD4 binding site antibodies neutralize autologous HIV-1. Cell Host Microbe 18:354–362. doi:10.1016/j.chom.2015.08.006. 26355218 PMC4567706

[30] Permar SR, Fong Y, Vandergrift N, Fouda GG, Gilbert P, Parks R, Jaeger FH, Pollara J, Martelli A, Liebl BE, Lloyd K, Yates NL, Overman RG, Shen X, Whitaker K, Chen H, Pritchett J, Solomon E, Friberg E, Marshall DJ, Whitesides JF, Gurley TC, Von Holle T, Martinez DR, Cai F, Kumar A, Xia SM, Lu X, Louzao R, Wilkes S, Datta S, Sarzotti-Kelsoe M, Liao HX, Ferrari G, Alam SM, Montefiori DC, Denny TN, Moody MA, Tomaras GD, Gao F, Haynes BF 2015 Maternal HIV-1 envelope-specific antibody responses and reduced risk of perinatal transmission. J Clin Invest 125:2702–2706 doi:10.1172/JCI81593. 26053661 PMC4613557

[31] Haynes BF, Gilbert PB, McElrath MJ, Zolla-Pazner S, Tomaras GD, Alam SM, Evans DT, Montefiori DC, Karnasuta C, Sutthent R, Liao H-X, DeVico AL, Lewis GK, Williams C, Pinter A, Fong Y, Janes H, DeCamp A, Huang Y, Rao M, Billings E, Karasavvas N, Robb ML, Ngauy V, de Souza MS, Paris R, Ferrari G, Bailer RT, Soderberg KA, Andrews C, Berman PW, Frahm N, De Rosa SC, Alpert MD, Yates NL, Shen X, Koup RA, Pitisuttithum P, Kaewkungwal J, Nitayaphan S, Rerks-Ngarm S, Michael NL, Kim JH 2012 Immune correlates analysis of the ALVAC-AIDSVAX HIV-1 vaccine efficacy trial. N Engl J Med 366:1275–1286. doi:10.1056/NEJMoa1113425. 22475592 PMC3371689

[32] Zolla-Pazner S, DeCamp AC, Gilbert PB, Williams C, Yates NL, Williams WT, Howington R, Fong Y, Morris DE, Soderberg KA, Irene C, Reichman C, Pinter A, Parks R, Pitisuttithum P, Kaewkungwal J, Rerks-Ngarm S, Nitayaphan S, Andrews C, O'Connell RJ, Yang Z-Y, Nabel GJ, Kim JH, Michael NL, Montefiori D, Liao H-X, Haynes BF, Tomaras G 2014 Vaccine-induced IgG antibodies to V1V2 regions of multiple HIV-1 subtypes correlate with decreased risk of HIV-1 infection. PLoS One 9:e87572. doi:10.1371/journal.pone.0087572. 24504509 PMC3913641

[33] Zolla-Pazner S, deCamp AC, Cardozo T, Karasavvas N, Gottardo R, Williams C, Morris DE, Tomaras G, Rao M, Billings E, Berman P, Shen X, Andrews C, O'Connell RJ, Ngauy V, Nitayaphan S, de Souza M, Korber B, Koup R, Bailer RT, Mascola JR, Pinter A, Montefiori D, Haynes BF, Robb ML, Rerks-Ngarm S, Michael NL, Gilbert PB, Kim JH 2013 Analysis of V2 antibody responses induced in vaccinees in the ALVAC/AIDSVAX HIV-1 vaccine efficacy trial. PLoS One 8:e53629. doi:10.1371/journal.pone.0053629. 23349725 PMC3547933

[34] Goudsmit J, Krone WJA, Wolfs TFW, Nara PL, Hartman S, Epstein LG, Tijnagel J 1989 Genomic divergence within the coding sequence for the principal neutralization epitope of HIV-1. Quatrieme Colloque Des Cent Gardes 4:55–60.

[35] Javaherian K, Langlois AJ, LaRosa GJ, Profy AT, Bolognesi DP, Herlihy WC, Putney SD, Matthews TJ 1990 Broadly neutralizing antibodies elicited by the hypervariable neutralizing determinant of HIV-1. Science 250:1590–1593. doi:10.1126/science.1703322. 1703322

[36] Zolla-Pazner S, Gorny MK, Nyambi PN 1999 The implications of antigenic diversity for vaccine development. Immunol Lett 66:159–164. doi:10.1016/S0165-2478(98)00176-X. 10203049

[37] Munro JB, Gorman J, Ma X, Zhou Z, Arthos J, Burton DR, Koff WC, Courter JR, Smith AB III, Kwong PD, Blanchard SC, Mothes W 2014 Conformational dynamics of single HIV-1 envelope trimers on the surface of native virions. Science 346:759–763. doi:10.1126/science.1254426. 25298114 PMC4304640

[38] Munro JB, Mothes W 2015 Structure and dynamics of the native HIV-1 Env trimer. J Virol 89:5752–5755. doi:10.1128/JVI.03187-14. 25762739 PMC4442439

[39] Sanders RW, Derking R, Cupo A, Julien JP, Yasmeen A, de Val N, Kim HJ, Blattner C, de la Pena AT, Korzun J, Golabek M, de Los Reyes K, Ketas TJ, van Gils MJ, King CR, Wilson IA, Ward AB, Klasse PJ, Moore JP 2013 A next-generation cleaved, soluble HIV-1 Env trimer, BG505 SOSIP.664 gp140, expresses multiple epitopes for broadly neutralizing but not non-neutralizing antibodies. PLoS Pathog 9:e1003618. doi:10.1371/journal.ppat.1003618. 24068931 PMC3777863

[40] Mao Y, Wang L, Gu C, Herschhorn A, Xiang S-H, Haim H, Yang X, Sodroski J 2012 Subunit organization of the membrane-bound HIV-1 envelope glycoprotein trimer. Nat Struct Mol Biol 19:893–899. doi:10.1038/nsmb.2351. 22864288 PMC3443289

[41] Xiang SH, Finzi A, Pacheco B, Alexander K, Yuan W, Rizzuto C, Huang CC, Kwong PD, Sodroski J 2010 A V3 loop-dependent gp120 element disrupted by CD4 binding stabilizes the human immunodeficiency virus envelope glycoprotein trimer. J Virol 84:3147–3161. doi:10.1128/JVI.02587-09. 20089638 PMC2838131

[42] Humes D, Emery S, Laws E, Overbaugh J 2012 A species-specific amino acid difference in the macaque CD4 receptor restricts replication by global circulating HIV-1 variants representing viruses from recent infection. J Virol 86:12472–12483. doi:10.1128/JVI.02176-12. 22973036 PMC3497638

[43] Humes D, Overbaugh J 2011 Adaptation of subtype A human immunodeficiency virus type 1 envelope to pig-tailed macaque cells. J Virol 85:4409–4420. doi:10.1128/JVI.02244-10. 21325401 PMC3126259

[44] Boyd DF, Peterson D, Haggarty BS, Jordan AP, Hogan MJ, Goo L, Hoxie JA, Overbaugh J 2015 Mutations in HIV-1 envelope that enhance entry with the macaque CD4 receptor alter antibody recognition by disrupting quaternary interactions within the trimer. J Virol 89:894–907. doi:10.1128/JVI.02680-14. 25378497 PMC4300673

[45] Long EM, Rainwater SM, Lavreys L, Mandaliya K, Overbaugh J 2002 HIV type 1 variants transmitted to women in Kenya require the CCR5 coreceptor for entry, regardless of the genetic complexity of the infecting virus. AIDS Res Hum Retroviruses 18:567–576. doi:10.1089/088922202753747914. 12036486

[46] Wu X, Parast AB, Richardson BA, Nduati R, John-Stewart G, Mbori-Ngacha D, Rainwater SM, Overbaugh J 2006 Neutralization escape variants of human immunodeficiency virus type 1 are transmitted from mother to infant. J Virol 80:835–844. doi:10.1128/JVI.80.2.835-844.2006. 16378985 PMC1346878

[47] Li M, Gao F, Mascola JR, Stamatatos L, Polonis VR, Koutsoukos M, Voss G, Goepfert P, Gilbert P, Greene KM, Bilska M, Kothe DL, Salazar-Gonzalez JF, Wei X, Decker JM, Hahn BH, Montefiori DC 2005 Human immunodeficiency virus type 1 env clones from acute and early subtype B infections for standardized assessments of vaccine-elicited neutralizing antibodies. J Virol 79:10108–10125. doi:10.1128/JVI.79.16.10108-10125.2005. 16051804 PMC1182643

[48] Klein F, Nogueira L, Nishimura Y, Phad G, West AP Jr, Halper-Stromberg A, Horwitz JA, Gazumyan A, Liu C, Eisenreich TR, Lehmann C, Fatkenheuer G, Williams C, Shingai M, Martin MA, Bjorkman PJ, Seaman MS, Zolla-Pazner S, Karlsson Hedestam GB, Nussenzweig MC 2014 Enhanced HIV-1 immunotherapy by naturally arising antibodies targeting resistant variants. J Exp Med 211:2361–2372. doi:10.1084/jem.20141050. 25385756 PMC4235636

[49] Gorny MK, Xu J-Y, Karwowska S, Buchbinder A, Zolla-Pazner S 1993 Repertoire of neutralizing human monoclonal antibodies specific for the V3 domain of HIV-1 gp120. J Immunol 150:635–643. 7678279

[50] Gorny MK, Williams C, Volsky B, Revesz K, Cohen S, Polonis VR, Honnen WJ, Kayman SC, Krachmarov CP, Pinter A, Zolla-Pazner S 2002 Human monoclonal antibodies specific for conformation-sensitive epitopes of V3 neutralize HIV-1 primary isolates from various clades. J Virol 76:9035–9045. doi:10.1128/JVI.76.18.9035-9045.2002. 12186887 PMC136433

[51] Gorny MK, Revesz K, Williams C, Volsky B, Louder MK, Anyangwe CA, Krachmarov CP, Kayman SC, Pinter A, Nadas A, Nyambi PN, Mascola JR, Zolla-Pazner S 2004 The V3 loop is accessible on the surface of most human immunodeficiency virus type 1 primary isolates and serves as a neutralization epitope. J Virol 78:2394–2404. doi:10.1128/JVI.78.5.2394-2404.2004. 14963135 PMC369230

[52] Gorny MK, Williams C, Volsky B, Revesz K, Wang XH, Burda S, Kimura T, Koning FA, Nadas A, Anyangwe C, Nyambi P, Krachmarov C, Pinter A, Zolla-Pazner S 2006 Cross-clade neutralizing activity of human anti-V3 monoclonal antibodies derived from the cells of individuals infected with non-B clades of HIV-1. J Virol 80:6865–6872. doi:10.1128/JVI.02202-05. 16809292 PMC1489067

[53] Gorny MK, Wang XH, Williams C, Volsky B, Revesz K, Witover B, Burda S, Urbanski M, Nyambi P, Krachmarov C, Pinter A, Zolla-Pazner S, Nadas A 2009 Preferential use of the VH5-51 gene segment by the human immune response to code for antibodies against the V3 domain of HIV-1. Mol Immunol 46:917–926. doi:10.1016/j.molimm.2008.09.005. 18952295 PMC2693011

[54] Gigler A, Dorsch S, Hemauer A, Williams C, Kim S, Young NS, Zolla-Pazner S, Wolf H, Gorny MK, Modrow S 1999 Generation of neutralizing human monoclonal antibodies against parvovirus B19 proteins. J Virol 73:1974–1979. 9971777 10.1128/jvi.73.3.1974-1979.1999 PMC104439

[55] Jiang X, Burke V, Totrov M, Williams C, Cardozo T, Gorny MK, Zolla-Pazner S, Kong X-P 2010 Conserved structural elements in the V3 crown of HIV-1 GP120. Nat Struct Mol Biol 17:955–961. doi:10.1038/nsmb.1861. 20622876

[56] Mayr LM, Zolla-Pazner S 2015 Antibodies targeting the envelope of HIV-1. Microbiol Spectr 3:AID-0025-2014. doi:10.1128/microbiolspec.AID-0025-2014. 26104552

[57] Li Y, Cleveland B, Klots I, Travis B, Richardson BA, Anderson D, Montefiori D, Polacino P, Hu SL 2008 Removal of a single N-linked glycan in human immunodeficiency virus type 1 gp120 results in an enhanced ability to induce neutralizing antibody responses. J Virol 82:638–651. doi:10.1128/JVI.01691-07. 17959660 PMC2224603

[58] O'Rourke SM, Schweighardt B, Phung P, Mesa KA, Vollrath AL, Tatsuno GP, To B, Sinangil F, Limoli K, Wrin T, Berman PW 2012 Sequences in glycoprotein gp41, the CD4 binding site, and the V2 domain regulate sensitivity and resistance of HIV-1 to broadly neutralizing antibodies. J Virol 86:12105–12114. doi:10.1128/JVI.01352-12. 22933284 PMC3486483

[59] Pinter A, Honnen WJ, He Y, Gorny MK, Zolla-Pazner S, Kayman SC 2004 The V1/V2 domain of gp120 is a global regulator of sensitivity of primary human immunodeficiency virus type 1 isolates to neutralization by antibodies commonly induced upon infection. J Virol 78:5205–5215. doi:10.1128/JVI.78.10.5205-5215.2004. 15113902 PMC400352

[60] Stamatatos L, Cheng-Mayer C 1998 An envelope modification that renders a primary, neutralization-resistant clade B human immunodeficiency virus type 1 isolate highly susceptible to neutralization by sera from other clades. J Virol 72:7840–7845. 9733820 10.1128/jvi.72.10.7840-7845.1998 PMC110102

[61] Bosch KA, Rainwater S, Jaoko W, Overbaugh J 2010 Temporal analysis of HIV envelope sequence evolution and antibody escape in a subtype A-infected individual with a broad neutralizing antibody response. Virology 398:115–124. doi:10.1016/j.virol.2009.11.032. 20034648 PMC2823950

[62] Kumar R, Pan R, Upadhyay C, Mayr LM, Cohen S, Wang X-H, Balasubramanian P, Nadas A, Seaman MS, Zolla-Pazner S, Gorny MK, Kong X-P, Hioe CE 2015 Functional and structural characterization of human V3-specific monoclonal antibody 2424 with neutralizing activity against HIV-1 JRFL. J Virol 89:9090–9102. doi:10.1128/JVI.01280-15. 26109728 PMC4524078

[63] McLellan JS, Pancera M, Carrico C, Gorman J, Julien JP, Khayat R, Louder R, Pejchal R, Sastry M, Dai K, O'Dell S, Patel N, Shahzad-ul Hussan S, Yang Y, Zhang B, Zhou T, Zhu J, Boyington JC, Chuang GY, Diwanji D, Georgiev I, Kwon YD, Lee D, Louder MK, Moquin S, Schmidt SD, Yang ZY, Bonsignori M, Crump JA, Kapiga SH, Sam NE, Haynes BF, Burton DR, Koff WC, Walker LM, Phogat S, Wyatt R, Orwenyo J, Wang LX, Arthos J, Bewley CA, Mascola JR, Nabel GJ, Schief WR, Ward AB, Wilson IA, Kwong PD 2011 Structure of HIV-1 gp120 V1/V2 domain with broadly neutralizing antibody PG9. Nature 480:336–343. doi:10.1038/nature10696. 22113616 PMC3406929

[64] Ringe R, Sharma D, Zolla-Pazner S, Phogat S, Risbud A, Thakar M, Paranjape R, Bhattacharya J 2011 A single amino acid substitution in the C4 region in gp120 confers enhanced neutralization of HIV-1 by modulating CD4 binding sites and V3 loop. Virology 418:123–132. doi:10.1016/j.virol.2011.07.015. 21851958 PMC4222521

[65] Horwitz JA, Halper-Stromberg A, Mouquet H, Gitlin AD, Tretiakova A, Eisenreich TR, Malbec M, Gravemann S, Billerbeck E, Dorner M, Buning H, Schwartz O, Knops E, Kaiser R, Seaman MS, Wilson JM, Rice CM, Ploss A, Bjorkman PJ, Klein F, Nussenzweig MC 2013 HIV-1 suppression and durable control by combining single broadly neutralizing antibodies and antiretroviral drugs in humanized mice. Proc Natl Acad Sci U S A 110:16538–16543. doi:10.1073/pnas.1315295110. 24043801 PMC3799352

[66] Li H, Xu CF, Blais S, Wan Q, Zhang HT, Landry SJ, Hioe CE 2009 Proximal glycans outside of the epitopes regulate the presentation of HIV-1 envelope gp120 helper epitopes. J Immunol 182:6369–6378. doi:10.4049/jimmunol.0804287. 19414790 PMC2808118

[67] O'Rourke SM, Schweighardt B, Phung P, Fonseca DP, Terry K, Wrin T, Sinangil F, Berman PW 2010 Mutation at a single position in the V2 domain of the HIV-1 envelope protein confers neutralization sensitivity to a highly neutralization-resistant virus. J Virol 84:11200–11209. doi:10.1128/JVI.00790-10. 20702624 PMC2953176

[68] Musich T, Peters PJ, Duenas-Decamp MJ, Gonzalez-Perez M, Robinson J, Zolla-Pazner S, Ball JK, Luzuriaga K, Clapham PR 2011 A conserved determinant in the V1 loop of HIV-1 that modulates the V3 loop to prime low CD4 use and macrophage infection. J Virol 85:2397–2405. doi:10.1128/JVI.02187-10. 21159865 PMC3067776

[69] Wei X, Decker JM, Wang S, Hui H, Kappes JC, Wu X, Salazar-Gonzalez JF, Salazar MG, Kilby JM, Saag MS, Komarova NL, Nowak MA, Hahn BH, Kwong PD, Shaw GM 2003 Antibody neutralization and escape by HIV-1. Nature 422:307–312. doi:10.1038/nature01470. 12646921

[70] Malenbaum SE, Yang D, Cavacini L, Posner M, Robinson J, Cheng-Mayer C 2000 The N-terminal V3 loop glycan modulates the interaction of clade A and B human immunodeficiency virus type 1 envelopes with CD4 and chemokine receptors. J Virol 74:11008–11016. doi:10.1128/JVI.74.23.11008-11016.2000. 11069996 PMC113181

[71] Koch M, Pancera M, Kwong PD, Kolchinsky P, Grundner C, Wang L, Hendrickson WA, Sodroski J, Wyatt R 2003 Structure-based, targeted deglycosylation of HIV-1 gp120 and effects on neutralization sensitivity and antibody recognition. Virology 313:387–400. doi:10.1016/S0042-6822(03)00294-0. 12954207

[72] Gorny MK, Stamatatos L, Volsky B, Revesz K, Williams C, Wang XH, Cohen S, Staudinger R, Zolla-Pazner S 2005 Identification of a new quaternary neutralizing epitope on human immunodeficiency virus type 1 virus particles. J Virol 79:5232–5237. doi:10.1128/JVI.79.8.5232-5237.2005. 15795308 PMC1069558

[73] Rong R, Bibollet-Ruche F, Mulenga J, Allen S, Blackwell JL, Derdeyn CA 2007 Role of V1V2 and other human immunodeficiency virus type 1 envelope domains in resistance to autologous neutralization during clade C infection. J Virol 81:1350–1359. doi:10.1128/JVI.01839-06. 17079307 PMC1797511

[74] Duenas-Decamp MJ, O'Connell O, Robinson J, Corti D, Zolla-Pazner S, Clapham PR 2012 The W100 pocket on HIV-1 gp120 is a unique target for the CD4bs monoclonal antibody b12. Retrovirology 9:9. doi:10.1186/1742-4690-9-9. 22284192 PMC3292835

[75] Wyatt R, Thali M, Tilley S, Pinter A, Posner M, Ho D, Robinson J, Sodroski J 1992 Relationship of the human immunodeficiency virus type 1 gp120 third variable loop to a component of the CD4 binding site in the fourth conserved region. J Virol 66:6997–7004. 1279195 10.1128/jvi.66.12.6997-7004.1992 PMC240347

[76] Totrov M 2014 Estimated secondary structure propensities within V1/V2 region of HIV gp120 are an important global antibody neutralization sensitivity determinant. PLoS One 9:e94002. doi:10.1371/journal.pone.0094002. 24705879 PMC3976368

[77] Korkut A, Hendrickson WA 2012 Structural plasticity and conformational transitions of HIV envelope glycoprotein gp120. PLoS One 7:e52170. doi:10.1371/journal.pone.0052170. 23300605 PMC3531394

[78] Guttman M, Cupo A, Julien JP, Sanders RW, Wilson IA, Moore JP, Lee KK 2015 Antibody potency relates to the ability to recognize the closed, pre-fusion form of HIV Env. Nat Commun 6:6144. doi:10.1038/ncomms7144. 25652336 PMC4338595

[79] Kwong PD, Doyle ML, Casper DJ, Cicala C, Leavitt SA, Majeed S, Steenbeke TD, Venturi M, Chaiken I, Fung M, Katinger H, Parren PW, Robinson J, Van Ryk D, Wang L, Burton DR, Freire E, Wyatt R, Sodroski J, Hendrickson WA, Arthos J 2002 HIV-1 evades antibody-mediated neutralization through conformational masking of receptor-binding sites. Nature 420:678–682. doi:10.1038/nature01188. 12478295

[80] Almond D, Cardozo T 2010 Assessment of immunologically relevant dynamic tertiary structural features of the HIV-1 V3 loop crown R2 sequence by ab initio folding. J Vis Exp 2010:e2118. doi:10.3791/2118. PMC3157871 20864931

[81] O'Rourke SM, Sutthent R, Phung P, Mesa KA, Frigon NL, To B, Horthongkham N, Limoli K, Wrin T, Berman PW 2015 Glycans flanking the hypervariable connecting peptide between the A and B strands of the V1/V2 domain of HIV-1 gp120 confer resistance to antibodies that neutralize CRF01_AE viruses. PLoS One 10:e0119608. doi:10.1371/journal.pone.0119608. 25793890 PMC4368187

[82] Goo L, Milligan C, Simonich CA, Nduati R, Overbaugh J 2012 Neutralizing antibody escape during HIV-1 mother-to-child transmission involves conformational masking of distal epitopes in envelope. J Virol 86:9566–9582. doi:10.1128/JVI.00953-12. 22740394 PMC3446598

[83] Bar KJ, Tsao CY, Iyer SS, Decker JM, Yang Y, Bonsignori M, Chen X, Hwang KK, Montefiori DC, Liao HX, Hraber P, Fischer W, Li H, Wang S, Sterrett S, Keele BF, Ganusov VV, Perelson AS, Korber BT, Georgiev I, McLellan JS, Pavlicek JW, Gao F, Haynes BF, Hahn BH, Kwong PD, Shaw GM 2012 Early low-titer neutralizing antibodies impede HIV-1 replication and select for virus escape. PLoS Pathog 8:e1002721. doi:10.1371/journal.ppat.1002721. 22693447 PMC3364956

[84] Rolland M, Edlefsen PT, Larsen BB, Tovanabutra S, Sanders-Buell E, Hertz T, deCamp AC, Carrico C, Menis S, Magaret CA, Ahmed H, Juraska M, Chen L, Konopa P, Nariya S, Stoddard JN, Wong K, Zhao H, Deng W, Maust BS, Bose M, Howell S, Bates A, Lazzaro M, O'Sullivan A, Lei E, Bradfield A, Ibitamuno G, Assawadarachai V, O'Connell RJ, deSouza MS, Nitayaphan S, Rerks-Ngarm S, Robb ML, McLellan JS, Georgiev I, Kwong PD, Carlson JM, Michael NL, Schief WR, Gilbert PB, Mullins JI, Kim JH 2012 Increased HIV-1 vaccine efficacy against viruses with genetic signatures in Env V2. Nature 490:417–420. doi:10.1038/nature11519. 22960785 PMC3551291

[85] Edlefsen PT, Rolland M, Hertz T, Tovanabutra S, Gartland AJ, deCamp AC, Magaret CA, Ahmed H, Gottardo R, Juraska M, McCoy C, Larsen BB, Sanders-Buell E, Carrico C, Menis S, Bose M, RV144 Sequencing Team, Arroyo MA, O'Connell RJ, Nitayaphan S, Pitisuttithum P, Kaewkungwal J, Rerks-Ngarm S, Robb ML, Kirys T, Georgiev IS, Kwong PD, Scheffler K, Pond SL, Carlson JM, Michael NL, Schief WR, Mullins JI, Kim JH, Gilbert PB 2015 Comprehensive sieve analysis of breakthrough HIV-1 sequences in the RV144 vaccine efficacy trial. PLoS Comput Biol 11:e1003973. doi:10.1371/journal.pcbi.1003973. 25646817 PMC4315437

[86] Walker LM, Phogat SK, Chan-Hui PY, Wagner D, Phung P, Goss JL, Wrin T, Simek MD, Fling S, Mitcham JL, Lehrman JK, Priddy FH, Olsen OA, Frey SM, Hammond PW, Miiro G, Serwanga J, Pozniak A, McPhee D, Manigart O, Mwananyanda L, Karita E, Inwoley A, Jaoko W, Dehovitz J, Bekker LG, Pitisuttithum P, Paris R, Allen S, Kaminsky S, Zamb T, Moyle M, Koff WC, Poignard P, Burton DR 2009 Broad and potent neutralizing antibodies from an African donor reveal a new HIV-1 vaccine target. Science 326:285–289. doi:10.1126/science.1178746. 19729618 PMC3335270

[87] Kong L, Wilson IA, Kwong PD 2015 Crystal structure of a fully glycosylated HIV-1 gp120 core reveals a stabilizing role for the glycan at Asn262. Proteins 83:590–596. doi:10.1002/prot.24747. 25546301 PMC4409329

[88] Shingai M, Nishimura Y, Klein F, Mouquet H, Donau OK, Plishka R, Buckler-White A, Seaman M, Piatak M Jr, Lifson JD, Dimitrov DS, Nussenzweig MC, Martin MA 2013 Antibody-mediated immunotherapy of macaques chronically infected with SHIV suppresses viraemia. Nature 503:277–280. doi:10.1038/nature12746. 24172896 PMC4133787

[89] Seaman MS, Janes H, Hawkins N, Grandpre LE, Devoy C, Giri A, Coffey RT, Harris L, Wood B, Daniels MG, Bhattacharya T, Lapedes A, Polonis VR, McCutchan FE, Gilbert PB, Self SG, Korber BT, Montefiori DC, Mascola JR 2010 Tiered categorization of a diverse panel of HIV-1 Env pseudoviruses for assessment of neutralizing antibodies. J Virol 84:1439–1452. doi:10.1128/JVI.02108-09. 19939925 PMC2812321

[90] Frey G, Chen J, Rits-Volloch S, Freeman MM, Zolla-Pazner S, Chen B 2010 Distinct conformational states of HIV-1 gp41 are recognized by neutralizing and non-neutralizing antibodies. Nat Struct Mol Biol 17:1486–1491. doi:10.1038/nsmb.1950. 21076402 PMC2997185

